# Enteroendocrine cells couple nutrient sensing to nutrient absorption by regulating ion transport

**DOI:** 10.1038/s41467-020-18536-z

**Published:** 2020-09-22

**Authors:** Heather A. McCauley, Andrea L. Matthis, Jacob R. Enriquez, Jonah T. Nichol, J. Guillermo Sanchez, William J. Stone, Nambirajan Sundaram, Michael A. Helmrath, Marshall H. Montrose, Eitaro Aihara, James M. Wells

**Affiliations:** 1 Division of Developmental Biology, Cincinnati Children’s Hospital Medical Center, 3333 Burnet Avenue, Cincinnati, OH 45229 USA; 2 Center for Stem Cell and Organoid Medicine, Cincinnati Children’s Hospital Medical Center, 3333 Burnet Avenue, Cincinnati, OH 45229 USA; 3 Department of Pharmacology and Systems Physiology, University of Cincinnati College of Medicine, 231 Albert Sabin Way, Cincinnati, OH 45267 USA; 4 Division of Pediatric General and Thoracic Surgery, Cincinnati Children’s Hospital Medical Center, 3333 Burnet Avenue, Cincinnati, OH 45229 USA; 5 Division of Endocrinology, Cincinnati Children’s Hospital Medical Center, 3333 Burnet Avenue, Cincinnati, OH 45229 USA

**Keywords:** Mechanisms of disease, Physiology, Gastrointestinal hormones, Gastrointestinal models

## Abstract

The ability to absorb ingested nutrients is an essential function of all metazoans and utilizes a wide array of nutrient transporters found on the absorptive enterocytes of the small intestine. A unique population of patients has previously been identified with severe congenital malabsorptive diarrhea upon ingestion of any enteral nutrition. The intestines of these patients are macroscopically normal, but lack enteroendocrine cells (EECs), suggesting an essential role for this rare population of nutrient-sensing cells in regulating macronutrient absorption. Here, we use human and mouse models of EEC deficiency to identify an unappreciated role for the EEC hormone peptide YY in regulating ion-coupled absorption of glucose and dipeptides. We find that peptide YY is required in the small intestine to maintain normal electrophysiology in the presence of vasoactive intestinal polypeptide, a potent stimulator of ion secretion classically produced by enteric neurons. Administration of peptide YY to EEC-deficient mice restores normal electrophysiology, improves glucose and peptide absorption, diminishes diarrhea and rescues postnatal survival. These data suggest that peptide YY is a key regulator of macronutrient absorption in the small intestine and may be a viable therapeutic option to treat patients with electrolyte imbalance and nutrient malabsorption.

## Abstract (web summary)

Enteroendocrine cells (EECs) are specialized gastrointestinal cells that have a role in nutrient sensing and hormone secretion. Here the authors show that peptide YY from EECs regulates nutrient absorption in intestinal organoids.

## Introduction

Enteroendocrine cells (EECs) are a rare population of cells found in the gastrointestinal epithelium that sense nutrients that are passing through the gut and in response secrete more than 20 distinct biologically active peptides. These peptides act in an endocrine or paracrine fashion to regulate all aspects of nutrient homeostasis including satiety, mechanical and chemical digestion, nutrient absorption, storage and utilization^1^. Humans^2^ and mice^3^ with genetic mutations that impact formation or function of EECs have intractable malabsorptive diarrhea, metabolic acidosis, and require parenteral nutrition or small-bowel transplant for survival. These findings were the first to link EECs to the absorption of macronutrients; however, the mechanism by which EECs contribute to this vital process is unknown. Poor absorption of macronutrients is a global health concern, with underlying etiology including short-gut syndrome, enteric pathogen infection, and malnutrition. Therefore, identification of factors regulating nutrient absorption has significant therapeutic potential.

Absorption of carbohydrate and protein requires coordinated activity of nutrient and ion transporters in the small intestine. Glucose is primarily absorbed via sodium-glucose cotransporter SGLT1, which uses a downhill Na^+^ gradient to transport one glucose or galactose molecule with two sodium ions from the lumen into the enterocyte^4^. The majority of dietary protein absorption occurs via Na^+^- and H^+^-linked amino acid transporters and PEPT1, which imports di- and tri-peptides coupled with a hydrogen ion^5^. The electrochemical gradients that drive nutrient absorption are maintained in part by ion transporters, including the cystic fibrosis transmembrane receptor (CFTR), which exports chloride^6^, and sodium-hydrogen exchanger NHE3, which maintains Na^+^ and H^+^ microclimates across the apical membrane^7^. Activity of CFTR and NHE3 are, in turn, regulated by levels of cyclic AMP (cAMP)^8,9^.

Most secreted EEC peptides signal via G protein-coupled receptors that act via second messenger cascade effectors like cAMP. Given the requirement for EECs in nutrient absorption, we investigated the possibility that EEC-derived peptides couple nutrient sensing to nutrient absorption by regulating electrogenic transport in neighboring enterocytes. Two well-studied peptides governing ion and water homeostasis in the colon are vasoactive intestinal peptide (VIP) and peptide YY (PYY). VIP, secreted from enteric neurons, signals via the G_αs_-coupled VIPR1 (VPAC1) on epithelial cells to raise levels of intracellular cAMP. In contrast, EEC-derived PYY acts in a paracrine fashion on colonocytes to lower cAMP via the epithelial G_αi_ coupled receptor NPY1R^10–13^. Moreover, PYY has been reported to augment postprandial nutrient absorption in the small intestine^14^. We posited that the mechanism underlying malabsorptive diarrhea in patients lacking EECs might be due to loss of EEC-ENS regulatory feedback in the small intestine, thus disrupting electrogenic nutrient absorption. Here, we find that PYY regulates normal ion transport and ion-coupled nutrient absorption in mouse and human small intestine, and that administration of exogenous PYY is sufficient to restore normal electrophysiology, nutrient absorption, and survival in EEC-deficient animals.

## Results

### The PYY-VIP axis regulates ion transport in small intestine

If EECs were required for regulating the normal electrophysiology of the small intestine, we would expect to see deranged ion transport in intestinal tissues lacking EECs. To investigate this, we used EEC-deficient mice (*VillinCre;Neurog3*^*flox/flox*^)^3^ and three different human small intestinal tissue models all derived from pluripotent stem cells (PSCs): human intestinal organoids (HIOs) derived in vitro^15^, HIOs that were matured to robust crypt-villus architecture in vivo^16^, and epithelial organoids (enteroids) derived from crypts of matured HIO tissues^16^. We generated EEC-deficient human small intestinal tissue by using PSC lines that had a null mutation in *NEUROG3*^17^, the basic helix-loop-helix transcription factor required for EEC formation in mice^18^ and humans^2^. As previously reported^19^, NEUROG3^−/−^ small intestinal organoids completely lacked EECs, but were otherwise normal in appearance (Supplementary Fig. 1).

**Fig. 1:**
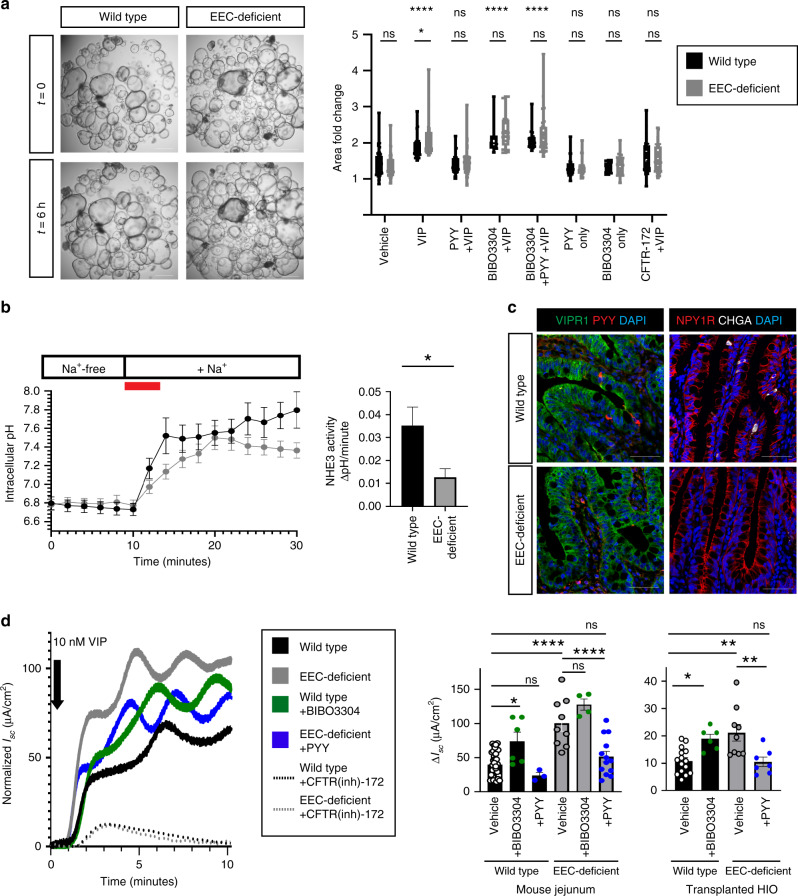
The PYY-VIP axis regulates ion and water transport in mouse and human small intestine. **a** PYY and VIP regulate ion and water transport in HIO-derived small intestinal enteroids. VIP-induced ion and water transport as measured by enteroid swelling (****P < 0.0001) in a CFTR-dependent manner. EEC-deficient enteroids had an elevated response to VIP compared to wild-type enteroids (*P = 0.04), which was inhibited in both cultures upon addition of PYY. Chemical inhibition of the PYY receptor NPY1R with BIBO3304 resulted in swelling of wild-type enteroids to EEC-deficient levels (****P < 0.0001) and abolished the inhibitory effects of PYY in both genotypes (****P < 0.0001). Scale bars = 500 μm. *n* = 283 wild-type, *n* = 351 EEC-deficient enteroids over three biologically independent lines. Statistics calculated by two-way ANOVA with Sidak’s multiple comparisons test; upper row indicates comparison to vehicle; lower row indicates comparison between wild-type and EEC-deficient. **b** EEC-deficient enteroids displayed impaired NHE3 activity. Quantification is of initial rate of Na^+^-dependent intracellular pH recovery (red line) after acid load using the ratiometric pH indicator SNARF-4F. *n* = 16 wild-type, *n* = 18 EEC-deficient enteroids; *P = 0.01; statistics calculated by unpaired, two-tailed Student’s *t* test. **c** The localization of VIPR1 and NPY1R was comparable between wild-type and EEC-deficient human intestinal epithelium. PYY+ and CHGA+ cells were only found in wild-type HIOs. Scale bars = 50 μm. Representative images from four independent organoids are shown. **d** PYY modulates the stimulatory effects of VIP in mouse and human small intestine. Using an Ussing chamber we observed EEC-deficient small intestinal tissue displayed a greater response (Δ*I*_sc_) to 10 nM VIP than wild-type (mouse, *n* = 20 wild-type, 8 mutant, ****P < 0.0001; human, *n* = 15 wild-type, 9 mutant, **P = 0.001). Inhibition of NPY1R in wild-type tissue with BIBO3304 resulted in an elevated response to VIP (mouse, *n* = 24, *P = 0.01; human, *n* = 7, *P = 0.04), whereas addition of exogenous PYY reduced the magnitude of EEC-deficient response to VIP (*n* = 8 mutant mice, ****P < 0.0001; *n* = 7 mutant HIOs, **P = 0.007) to wild-type levels. Electrogenic responses to VIP were blocked by the CFTR inhibitor CFTR-172 (dotted lines). One representative trace is shown (mouse), with baseline *I*_sc_ normalized to 0 μA/cm^2^. Statistics calculated by one-way ANOVA with Tukey’s multiple comparisons test. All error bars are + SEM.

In the colon, ion and water transport is regulated by EEC-derived PYY and ENS-derived VIP. To formally test whether the PYY-VIP axis also operated in human and mouse small intestine, we performed experiments in EEC-deficient tissues without a functional ENS wherein we controlled PYY and VIP levels experimentally. We first determined the effects of the PYY-VIP axis on small intestine by measuring CFTR-mediated ion and water efflux^20^ following exposure of human HIO-derived enteroids to the potent secretagogue VIP (Fig. 1a). EEC-deficient enteroids swelled significantly more than wild-type, but blocking the PYY receptor NPY1R in wild-type enteroids mimicked the EEC-deficient response (Fig. 1a). Exogenous PYY blocked VIP-induced swelling in both wild-type and EEC-deficient enteroids in an NPY1R-dependent manner (Fig. 1a), demonstrating that the PYY-VIP axis regulates ion and water secretion in human small intestine. Next, we tested the activity of the Na^+^/H^+^ exchanger NHE3. We cultured enteroids in a ratiometric pH-sensitive dye and manipulated the media surrounding the enteroids to remove all Na^+^ while simultaneously acid-loading the cells. When Na^+^-containing media was added back to the enteroids, NHE3 function could be measured as recovery of intracellular pH^21^. In this assay, we observed that EEC-deficient enteroids displayed impaired NHE3 function (Fig. 1b). There was no difference in expression of *CFTR, SLC9A3* (encoding NHE3), *VIPR1* or *NPY1R* between wild-type and EEC-deficient human small intestinal organoids or enteroids (Fig. 1c and Supplementary Fig. 2a-b). Together, these data suggest that PYY plays an important role in the regulation of ion transport in the small intestine, and that the abnormal response to VIP in EEC-deficient enteroids can be normalized by the addition of exogenous PYY.

If PYY were required to regulate electrochemical transport in the small intestine, we would expect that disruption of PYY signaling in wild-type small intestinal tissue would cause abnormal basal short-circuit current (*I*_sc_). To investigate this, we isolated full thickness intestinal mucosa from in vivo matured HIOs and from the jejunum of wild-type mice and measured basal *I*_sc_ in a modified Ussing chamber^22^. Chemical inhibition of NPY1R in wild-type mouse jejunum and HIOs was sufficient to elevate the basal *I*_*sc*_ to EEC-deficient levels (Supplementary Fig. 2c). Conversely, treatment of EEC-deficient mouse and human tissues with exogenous PYY reduced the basal *I*_*sc*_ to wild-type levels in an NPY1R-dependent manner (Supplementary Fig. 2c). These data indicated that endogenous PYY signaling plays an essential role in maintaining normal electrophysiology in the small intestine.

We then investigated if PYY was required to modulate the stimulatory effects of VIP in mouse and human small intestine. We inhibited voltage-gated neuronal firing in mouse jejunum by including tetrodotoxin^10^ in all experiments so that we could precisely monitor epithelial response to exogenous VIP. Chemical inhibition of NPY1R in isolated wild-type tissues was sufficient to cause an elevated response to VIP (Fig. 1d). This indicated that endogenous PYY signaling was required in the small intestine to modulate the stimulatory effects of VIP. Consistent with this, EEC-deficient mouse and human small intestinal tissue similarly displayed an exaggerated *I*_sc_ response to exogenous VIP compared to wild-type (Fig. 1d). Addition of exogenous PYY to EEC-deficient small intestine was sufficient to restore the *I*_sc_ to normal (Fig. 1d). These data suggested that PYY is required for maintaining a normal electrochemical response to VIP in the small intestine and that exogenous PYY can normalize this process in EEC-deficient small intestinal tissue. Furthermore, these data suggest that imbalance of this axis may be a mechanism underlying electrolyte imbalance, diarrhea and poor nutrient absorption suffered by patients without EECs.

### PYY regulates glucose absorption in small intestine

**Fig. 2:**
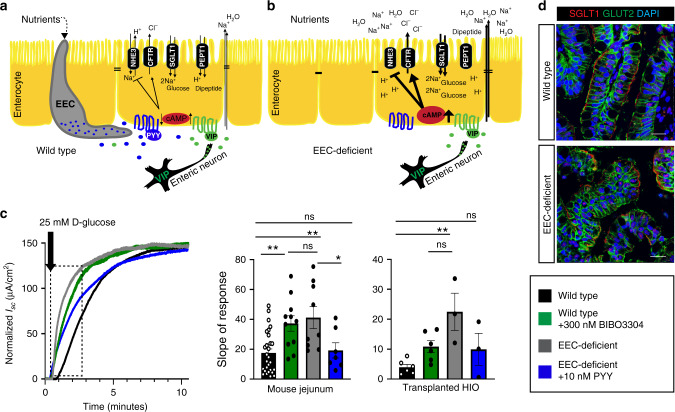
PYY restores normal glucose absorption in EEC-deficient human and mouse small intestine. **a** Schematic depicting how the PYY-VIP paracrine axis might regulate ion, water, and nutrient transport in the small intestine. **b** In the absence of EECs, ion, water, and nutrient transport are dysregulated due to loss of one arm of the PYY-VIP axis. In EEC-deficient small intestine, loss of PYY results in increased chloride transport and increased water and sodium accumulation in the intestinal lumen. Reduced NHE3 transport activity would cause accumulation of cytosolic H^+^ and a decrease in pH. Subsequently, nutrient absorption would be dysregulated, with diminished di-/tri-peptide absorption due to increased intracellular proton accumulation and increased uptake of glucose due to an exaggerated Na^+^ gradient across the apical membrane. **c** Na^+^-coupled glucose transport is deranged in EEC-deficient human and mouse small intestine. Wild-type and EEC-deficient human and mouse intestinal tissues were treated with VIP prior to 25 mM D-glucose. EEC-deficient intestine had an elevated initial response to glucose (mouse, *n* = 28 wild-type, *n* = 9 mutant, **P = 0.001; HIO, *n* = 6 wild-type, *n* = 4 mutant, **P = 0.002) that was returned to wild-type levels by pre-treatment with 10 nM exogenous PYY (mouse, *n* = 7, *P = 0.04; HIO, *n* = 3). Inhibition of NPY1R in wild-type tissues using BIBO3304 caused an abnormal initial response to glucose that mimicked EEC-deficient tissues (mouse, *n* = 12, **P = 0.005; HIO, *n* = 6). Bar graphs represent the slope of the curve depicted within the boxed area. Statistics calculated by one-way ANOVA with Tukey’s multiple comparisons test. **d** The subcellular distribution of glucose transporters SGLT1 and GLUT2 is normal in human intestinal tissue lacking EECs. Representative images from eight independent organoids are shown. Scale bars = 50 μm. All error bars are + SEM.

While it is known that EECs sense nutrients, the mechanism linking sensing to the control of nutrient absorption is unclear. A hint came from the effects of enteral feeding of EEC-deficient patients, which resulted in a massive diarrheal response. This suggests that an inability to sense luminal nutrients uncoupled the ability to adequately absorb them. To explore this possibility, we evaluated ion-coupled nutrient absorption in EEC-deficient small intestine. We observed an accelerated initial response to luminal glucose in the presence of VIP in EEC-deficient mouse and human intestinal tissues in the Ussing chamber (Fig. 2c), as predicted if the normal electrochemical gradients were perturbed (Fig. 2a-b). This recapitulated the exacerbated diarrhea observed in patients without EECs when they were fed with carbohydrate^2^. Exogenous PYY restored a normal glucose response in EEC-deficient mouse and human tissue, and inhibition of NPY1R in wild-type caused an exaggerated initial response to glucose (Fig. 2c). These data indicate that PYY is both necessary and sufficient to modulate glucose absorption in the small intestine. We found no defects in expression of SGLT1, GLUT2, (Fig. 2d and Supplementary Fig. 2a) or maximum absorptive competency of Na^+^-coupled glucose transport (Supplementary Fig. 3) in human epithelium without EECs. These data suggest that SGLT1 is competent to absorb glucose, but activity is dysregulated in the context of abnormal ion transport in the absence of EECs.

### Dipeptide absorption is impaired in the absence of EECs

**Fig. 3:**
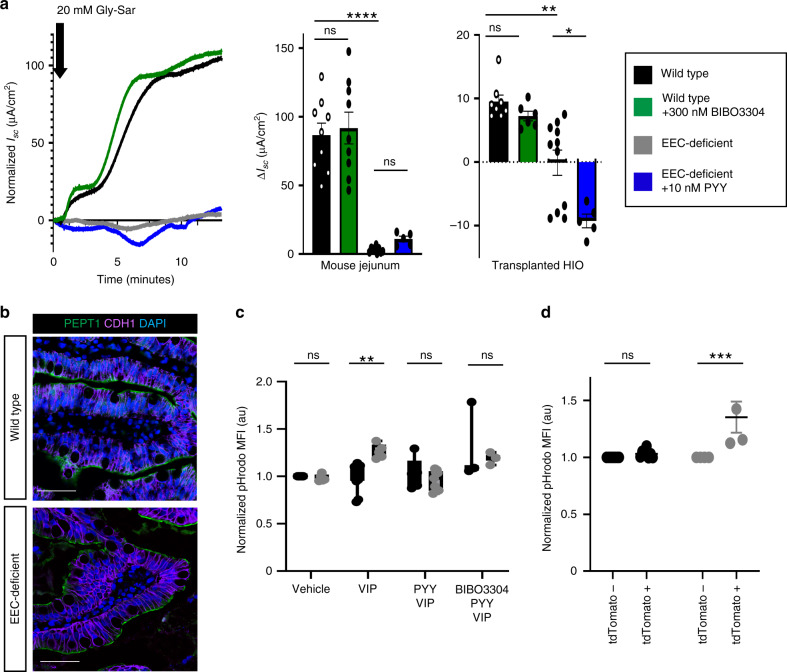
H^+^-coupled dipeptide absorption is impaired in EEC-deficient small intestine. **a** EEC-deficient human and mouse small intestine did not respond to the dipeptide Gly-Sar after exposure to VIP (mouse, *n* = 9 wild-type, *n* = 6 mutant, ****P < 0.0001; human, *n* = 11 wild-type, *n* = 5 mutant, **P = 0.006). Pre-treatment of EEC-deficient tissue with exogenous PYY (mouse, *n* = 6, ns; human, *n* = 5, *P = 0.03), or of wild-type tissue with BIBO3304 (mouse, *n* = 9; human, *n* = 6) did not improve the *I*_sc_ response to Gly-Sar. Statistics calculated by one-way ANOVA with Tukey’s multiple comparisons test. **b** Expression and localization of peptide transporter PEPT1 is unchanged in EEC-deficient human small intestine. Representative images from eight independent organoids are shown. Scale bars = 50 μm. **c** The PYY-VIP axis regulates intracellular pH in human small intestinal cells. EEC-deficient enteroids differentiated with VIP for 5–7 days developed an H^+^ imbalance with an acidic cytoplasm whereas wild-type enteroids were able to maintain their intracellular pH (**P = 0.004). Concurrent treatment with 10 nM PYY normalized the pH in EEC-deficient enteroids and was dependent on NPY1R. pHrodo MFI was analyzed by flow cytometry and normalized to vehicle-treated wild-type. *n* = 3 independent experiments. Statistics calculated by mixed effects analysis using the Holm–Sidak method. **d** Small intestinal EECs regulate proton transport in a paracrine fashion. Using reporter animals with mosaic loss of EECs we found that regions of jejunal epithelium that escaped recombination had normal pH as measured by pHrodo MFI. Adjacent regions that expressed tdTomato exhibited increased pHrodo MFI, indicating elevated cytosolic H^+^ (*n* = 4 mice, ***P = 0.0002). There was no difference in pHrodo MFI between mosaic regions in wild-type reporter jejunum (*n* = 8 mice). Statistics calculated by two-way ANOVA with Sidak’s multiple comparisons test. All error bars are + SEM.

Approximately 80% of ingested amino acids were recovered in the stool of the index EEC*-*deficient patient^2^, suggesting a critical role for EECs in regulating protein absorption. Consistent with this, we observed a striking loss of ion-coupled dipeptide absorption when human and mouse EEC-deficient small intestine were challenged with VIP (Fig. 3a), despite normal expression of PEPT1 and equivalent dipeptide absorption in vitro (Fig. 3b and Supplementary Figs. 2a and 4). VIP has an established role in inhibition of NHE3 and PEPT1-mediated dipeptide absorption^7,23^, but we were surprised to find that EEC-deficient intestine remained unable to respond to dipeptide when PYY was provided (Fig. 3a). This suggested that dysregulated H^+^ gradients may be a more stable phenotype in EEC-deficient intestine, and not easily reversed by PYY within minutes. To explore this possibility, we treated enteroids with or without PYY for 1 week in vitro in the presence of VIP. Wild-type enteroids were able to maintain their intracellular pH in the presence of VIP but EEC-deficient enteroids became significantly more acidic (Fig. 3c). However, EEC-deficient enteroids were restored to normal intracellular pH levels and normal *SLC9A3* expression (encoding NHE3) in the presence of PYY (Fig. 3c and Supplementary Fig. 5). This suggested that long-term exposure to an imbalanced EEC-ENS axis dysregulates intestinal physiology, and that, over time, PYY may be sufficient to restore intracellular pH and dipeptide absorption in EEC-deficient small intestine.

We have demonstrated that inhibiting PYY signaling in isolated wild-type small intestinal tissues was sufficient to perturb normal electrophysiology in both human and mouse. This suggests that in vivo the mechanism of action of PYY could be paracrine rather than endocrine. PYY-expressing EECs are abundant in mouse and human small intestine^24^ (Supplementary Fig. 6). Moreover, PYY-expressing EECs extend long basal processes which underlie several neighboring epithelial cells^25,26^, raising the possibility that they may exert paracrine effects on whole populations of nearby enterocytes. We therefore investigated whether the effects of PYY on ion transport in the small intestine occurred via paracrine mechanisms. To do this, we exploited the mosaicism of *VillinCre* mice to determine if regions of EEC-deficient epithelium had different transporter activities as compared to regions of epithelium that still had EECs. We crossed *VillinCre;Neurog3*^*+/+*^ and *VillinCre;Neurog3*^*flox/flox*^ mice to the *Rosa26*^*flox−stop−flox−tdTomato*^ reporter mouse to track regions of epithelium which had successfully recombined. We observed in *VillinCre;Neurog3*^*flox/flox*^ mice that 4.38 + 2.56% of jejunum escaped tdTomato labeling (Supplementary Fig. 7) and that in regions that had EECs, neighboring enterocytes had a normal intracellular pH indicating normal ion transport. In contrast, enterocytes in EEC-deficient regions were significantly more acidic indicating perturbed H^+^ transport (Fig. 3d and Supplementary Fig. 7). Together these data suggest that EECs control local H^+^ transporter activity and dipeptide responsiveness in the small intestine via paracrine mechanisms.

### Exogenous PYY rescues phenotypes of EEC-deficient mice

**Fig. 4:**
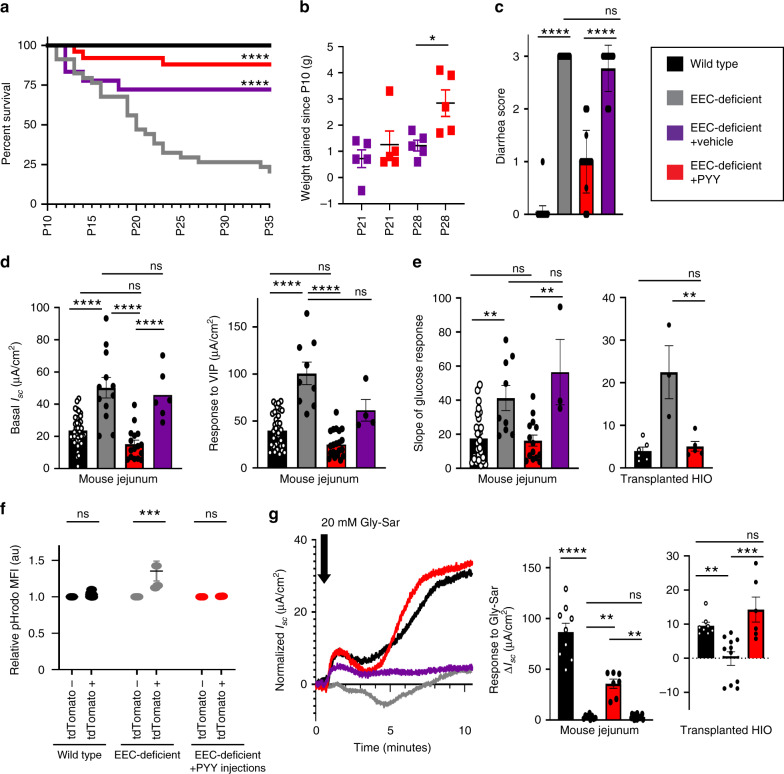
Exogenous PYY rescues EEC-deficient mice from malabsorptive diarrhea and restores normal glucose and dipeptide transport. **a** Survival curve of wild-type mice (*n* = 100), EEC-deficient mice (*n* = 34), EEC-deficient mice treated once daily with 10 μg PYY (*n* = 25) beginning at postnatal day 10 (P10), and vehicle-treated EEC-deficient mice (*n* = 18). ****P < 0.0001 comparison of survival curves to untreated mutant by log-rank Mantel–Cox test. **b** At postnatal day 28, PYY-treated mutant mice experienced significant weight gain compared to vehicle-treated mutant mice (*P = 0.01, *n* = 5 mice per condition). Statistics calculated by one-way ANOVA. **c** EEC-deficient mice have intractable watery diarrhea from birth (*n* = 34 mice; ****P < 0.0001 from wild-type littermates, *n* = 100 mice). Within 48 h of PYY treatment, EEC-deficient animals improved to slightly soft yet well-defined fecal pellets (*n* = 25 mice, ****P < 0.0001 from untreated mutant). Vehicle-treated mutant mice did not improve (*n* = 18 mice). Statistics calculated by one-way ANOVA with Tukey’s multiple comparisons test. **d** PYY treatment of EEC-deficient animals restored a normal resting *I*_sc_ to small intestine (*n* = 6 mice, ****P < 0.0001) and a normal electrogenic response to VIP (*n* = 6 mice, ****P < 0.0001). Treatment of mutant mice with vehicle did not improve basal *I*_sc_ (*n* = 6 mice) or response to VIP (*n* = 4 mice). Statistics calculated by one-way ANOVA with Tukey’s multiple comparisons test. **e** PYY treatment restored a normal glucose response in EEC-deficient mouse and human intestine (mouse, *n* = 6, **P = 0.003; HIO, *n* = 5, **P = 0.004). Statistics calculated by one-way ANOVA with Tukey’s multiple comparisons test. **f** Proton transport, as measured by pHrodo MFI, was normalized between mosaic regions in EEC-deficient reporter animals following PYY treatment (*n* = 2 mice). Statistics calculated by two-way ANOVA with Sidak’s multiple comparisons test. **g** PYY improved dipeptide transport in EEC-deficient mouse and human intestine. Long-term treatment of EEC-deficient animals and transplanted HIOs with PYY resulted in improved *I*_sc_ response to Gly-Sar compared to untreated mutant tissue (mouse, *n* = 6, **P = 0.009; HIO, *n* = 5, ***P = 0.0001). Vehicle-treated mutant mice did not exhibit improvement in Gly-Sar response (*n* = 18 mice, **P = 0.002). Statistics calculated by one-way ANOVA with Tukey’s multiple comparisons test. All wild-type and untreated mutant mouse data points are the same as shown in Figs. 1–3, and S2. All error bars are + SEM.

As previously reported^3^, *VillinCre;Neurog3*^*flox/flox*^ mice suffer from malabsorptive diarrhea and exhibit severely impaired postnatal survival, with only a small fraction of mice surviving weaning. Our data suggested that treatment with PYY might restore normal carbohydrate and protein absorption in the intestines of EEC-deficient animals. We therefore used *VillinCre;Neurog3*^*flox/flox*^ mice as a preclinical model to test if PYY could reverse malabsorptive diarrhea and improve postnatal survival (Fig. 4). We began daily treatment of mutant mice at postnatal day 10 with 10 μg PYY(1–36) by intraperitoneal injection. PYY can be converted to PYY(3–36) by the protease DPP4^27^, and this form of PYY has potent anorexic effects in the brain^28^. We therefore co-injected PYY(1–36) and a DPP4 inhibitor to prevent PYY cleavage and to better target the epithelial NPY1R receptor that preferentially binds the 1–36 form^10,12,27^. We simultaneously treated another group of mutant mice with vehicle, DPP4 inhibitor diluted in water. Patients with EEC-deficiency die without total parenteral nutrition, and similarly very few EEC-deficient mice survive without treatment within the first few weeks. Treatment of mutant mice with vehicle or with PYY significantly improved survival, consistent with therapeutic administration of supportive fluids in diarrheal disease (Fig. 4a). However, only PYY injections helped animals gain body weight (Fig. 4b), suggesting improvements in nutrient absorption. PYY also resulted in reduced diarrhea and improved fecal output to be nearly indistinguishable from wild-type, which was independent of intestinal motility (Fig. 4c and Supplementary Fig. 8).

PYY has a well-established anti-secretory role in the colon, which was likely an important factor contributing to the improvement in diarrheal symptoms observed in EEC-deficient mice. However, our data suggested that PYY may have additional roles in nutrient and ion transport in the small intestine. Therefore, we investigated if the animals that received PYY injections had restored electrophysiology and improved nutrient absorption in the small intestine. We found that PYY injections restored the basal *I*_sc_ of jejunum to normal (Fig. 4d). In addition, the response to VIP (Fig. 4d) and the response to luminal glucose (Fig. 4e) were both normalized indicating that PYY injections stably restored electrophysiology. Importantly, mice received their last injection of PYY ~16 h prior to sacrifice, demonstrating sustained action of the peptide in vivo. The rescue of EEC-deficient intestinal tissue also extended to the human model, where EEC-deficient HIOs were grown and matured in vivo and then host animals were injected with exogenous PYY for 10 days prior to harvest. These EEC-deficient HIOs exposed to PYY demonstrated electrogenic response to glucose that was indistinguishable from wild-type (Fig. 4e). Lastly, we investigated whether the PYY treated groups had improved amino acid absorption as measured by H^+^ export and response to the dipeptide Gly-Sar. By administering PYY to the mosaic EEC-deficient reporter mice, we found PYY injections restored intracellular pH in EEC-deficient intestinal cells to normal levels which would support PEPT1-mediated dipeptide absorption (Fig. 4f). Consistent with this, PYY-injected mouse and human small intestine displayed a significantly improved electrogenic response to dipeptides (Fig. 4g), indicating that dipeptide absorption was restored. These data demonstrated functional efficacy of PYY on improved ion and nutrient transport in EEC-deficient intestine.

## Discussion

In this study, we found that loss of all EECs resulted in profound imbalance of ion transport in the small intestine, with subsequent impairment of nutrient absorption. We demonstrated that the peptide hormone PYY functions in the small intestine to regulate normal electrophysiology and absorption. Chemical inhibition of the epithelial NPY1R receptor in wild-type small intestine isolated from HIOs and mouse demonstrated the requirement of this pathway in the modulation of VIP-induced ion secretion. Administration of PYY to EEC-deficient animals resulted in improvements in survival, diarrheal symptoms, glucose absorption, and protein absorption in the absence of all other EEC peptides.

Historically, mouse models have been exceedingly tolerant of loss of individual EEC populations, largely due to functional overlap between EEC-derived peptides^29^. This has rendered it difficult to assign roles of individual EEC peptides to physiologic functions. Here, we were able to exploit a model which lacks all EECs to functionally evaluate the role of one EEC peptide, PYY. However, other peptides like somatostatin have similar activities to PYY and likely play a similar regulatory role in vivo. Somatostatin has many systemic targets^30^ and the use of the somatostatin-analogue octreotide in the treatment of chylous effusion and hyperinsulinemia causes an increased risk of necrotizing enterocolitis in infants^31^. We therefore chose to use PYY in our preclinical model of ion-coupled nutrient absorption and diarrhea.

PYY has been classically defined as a satiety hormone that acts in an endocrine manner wherein the DPP4-cleaved PYY(3–36) signals to the brain to reduce food intake^28^. However PYY(1–36) has been shown to act in a paracrine manner in the colon using combination of genetic and pharmacological approaches^10,12,32^. We and others^24^ observe abundant PYY+ cells in the small bowel, suggesting that these cells may also have a paracrine role in the small intestine to regulate ion and water transport that is linked to glucose and protein absorption. These findings lend some clarity on how EECs integrate their nutrient sensing function with nutrient absorption, providing us with a new way to approach management of absorptive diseases and those in which EECs are commonly dysregulated.

## Methods

### Pluripotent stem cell culture and directed differentiation of HIOs

Human embryonic stem cell (ESC) line WA01 (H1) was purchased from WiCell. We used H1 cells with a CRISPR/Cas9 generated null mutation in *NEUROG3*^17^. In addition, we inserted the CDH1-mRuby2 reporter construct^33^ into *NEUROG3-/-* H1 hESCs. CDH1-mRuby2 and non-reporter hESCs were used interchangeably. hESCs were maintained in feeder-free culture. Cells were plated on hESC-qualified Matrigel (BD Biosciences, San Jose, CA) and maintained at 37 °C with 5% CO_2_ with daily removal of differentiated cells and replacement of mTeSR1 media (STEMCELL Technologies, Vancouver, Canada). Cells were passaged routinely every 4 days using Dispase (STEMCELL Technologies). HIOs were generated according to protocols established in our lab^15,34^ and experiments with human ESCs were approved by the Cincinnati Children’s Hospital ESCRO committee (Protocol #EIPDB2713).

### In vivo transplant of HIOs

28–35 days after spheroid generation, HIOs were removed from Matrigel and transplanted under the kidney capsule of immune deficient NOD.Cg-*Prkdc*^*scid*^*Il2rg*^*tm1Wjl*^/SzJ (NSG) mice^16^. NSG mice were maintained on Bactrim chow for a minimum of 2 weeks prior to transplantation and thereafter for the duration of the experiment (8–14 weeks).

### Generation and maintenance of HIO-derived enteroids

After ~10 weeks of in vivo growth, crypts were isolated from transplanted HIOs and plated in 3D^35^. To promote growth, enteroids were maintained in Human IntestiCult components A + B (STEMCELL Technologies). To promote differentiation, enteroids were cultured in gut media^34^ with 100 μg/ml EGF for 5–7 days. Undifferentiated enteroids were passaged every 7–10 days into fresh Matrigel (Corning) using a 25 G ×1/2 needle.

### Immunofluorescence

Tissue was fixed in 4% paraformaldehyde, cryopreserved in 30% sucrose, embedded in OCT, and frozen at −80 °C until cryosectioned. 8 μm cryosections were mounted on Superfrost Plus slides and permeabilized, blocked, and stained according to standard protocol. Primary antibodies used are listed in the table below, and all secondary antibodies were conjugated to Alexa Fluor 488, 546/555/568 or 647 (Invitrogen) and used at 1:500 dilution. Images were acquired using a Nikon A1 GaAsP LUNV inverted confocal microscope and NIS Elements software (Nikon).

**Table:** 

*Primary antibody*	*Company*	*Host*	*Dilution*
CDX2	BioGenex	Mouse	1:300
CDX2	Cell Marquis	Rabbit	1:500
Chromogranin A	DSHB	Mouse	1:500
Chromogranin A	ImmunoStar	Rabbit	1:250
E-Cadherin (CDH1)	R&D	Goat	1:500
GLUT2	Santa Cruz	Goat	1:500
Muc2	Santa Cruz	Rabbit	1:250
NPY1R	Abcam	Rabbit	1:250
PDX1	Abcam	goat	1:5000
PEPT1	Santa Cruz	Rabbit	1:500
PYY	Abcam	Rabbit	1:1000
SGLT1	Santa Cruz	Rabbit	1:250
Somatostatin	Santa Cruz	Goat	1:200
VIPR1	ThermoFisher Scientific	Rabbit	1:200

### qPCR

RNA was extracted using the Nucleospin RNA extraction kit (Macharey-Nagel) and reverse transcribed into cDNA using Superscript VILO (Invitrogen) according to manufacturer’s instruction. qPCR primers were designed using NCBI PrimerBlast. Primer sequences are listed in the table below. qPCR was performed using Quantitect SYBR^®^ Green PCR kit (QIAGEN) and a QuantStudio 3 Flex Real-Time PCR System (Applied Biosystems). Relative expression was determined using the ΔΔCt method and normalizing to PPIA (cyclophilin A). Samples from at least three independent passages were used for quantification.

**Table:** 

PPIA (CPHA) FWD	CCCACCGTGTTCTTCGACATT
PPIA (CPHA) REV	GGACCCGTATGCTTTAGGATGA
CHGA FWD	TGTGTCGGAGATGACCTCAA
CHGA REV	GTCCTGGCTCTTCTGCTCTG
PYY FWD	CGAGACTAAATGTGGCGGGT
PYY REV	GAGCATGCAGTTCTGAGGGT
SST FWD	TGGGTTCAGACAGCAGCTC
SST REV	CCCAGACTCCGTCAGTTTCT
VIP FWD	CCCTGTACCAGTCAAACGTCA
VIP REV	GAGTCTCCATGCAGGCTTCT
PDX1 FWD	CGTCCGCTTGTTCTCCTC
PDX1 REV	CCTTTCCCATGGATGAAGTC
CDX2 FWD	GGGCTCTCTGAGAGGCAGGT
CDX2 REV	GGTGACGGTGGGGTTTAGCA
NPY1R FWD	ATTCCTAGGCAATGCTTCCCC
NPY1R REV	ACGCCTCCTTAAAGCCGAAC
VIPR1 FWD	GATAGGAGCCTGCTGGTCAC
VIPR1 REV	GGGGAACCAAGCCAATCCAA
CFTR FWD	GGCACCCAGAGTAGTAGGTC
CFTR REV	AGGCGCTGTCTGTATCCTTT
SLC9A3 (NHE3) FWD	GCTGGTCTTCATCTCCGTGT
SLC9A3 (NHE3) REV	CCAGAGGCTTGATGGTCAGG

### Swelling assay

Enteroids were plated in 10 μL Matrigel on an 8-chamber glass bottom slide (Ibidi) and maintained as described above. 3–5 days post-plating, the slide was mounted on an inverted confocal microscope (Nikon) fitted with an incubation chamber set to 37 °C and 5% CO_2_. Media was changed to include 10 nM VIP (Tocris). In some experiments, the media was changed 24 h prior to imaging to include 300 nM BIBO3304 trifluoroacetate (Tocris), 20 μM CFTR(inh)−172 (Millipore Sigma) and/or 10 nM PYY (Phoenix Pharmaceuticals). Images were acquired every 5 min at ×4 magnification. After 6 h, some HIOEs swelled to the point of bursting; therefore, we used images acquired at time 0 and at 6 h for quantification. The area of ten representative enteroids per well was quantified using NIS Elements software at both time points. The outline of individual enteroids was traced manually and the area calculated by NIS Elements. Fold change at 6 h over baseline was reported. Data include a minimum of three independent experiments per condition on three wild-type and three EEC-deficient HIO-derived enteroid lines.

### NHE3 activity assay

NHE3 activity was determined by confocal live imaging of enteroids with a ratiometric pH-sensitive dye^21^. Enteroids were plated in 5 μL Matrigel on an 8-chamber glass bottom slide (Ibidi) and maintained as described above. 3–5 days post-plating, media was changed to Na^+^ media containing 5 μM SNARF-4F 5-(and-6)- carboxylic acid, acetoxymethyl ester, acetate (Molecular Probes) and allowed to incubate for 30 min. The slide was then mounted on an inverted confocal microscope (Nikon), fitted with an incubation chamber set to 37 °C and 5% CO_2_. Fresh Na^+^ media was provided before image acquisition. Images were acquired every 2 min for 2 h at 10X magnification with excitation at 488 nm and emission at 561 nm and 640 nm. Media was changed to NH_4_Cl to acid-load the epithelium, then to tetramethylammonium (TMA) media to withdraw Na^+^. Na^+^-containing media was then added and NHE3 activity quantified as a measure of initial pH recovery. 1 mM probenecid and 5 μM SNARF were present in all buffers, and all buffers were set to pH 7.4. Intracellular pH was calibrated using the Intracellular pH Calibration Buffer kit (Invitrogen) at pH 7.5, 6.5, and 5.5 in the presence of 10 μM valinomycin and 10 μM nigericin at the conclusion of each experiment. The ratio of 561/640 was determined using NIS Elements software by drawing a region of interest and quantifying the fluorescence intensity of each wavelength over the period of the experiment. A minimum of three enteroids in three wells over two independent passages were quantified. The ratio of 561/640 was converted to intracellular pH using the equation provided by the manufacturer.

Na^+^ media: 130 mM NaCl, 5 mM KCl, 2 mM CaCl_2_, 1 mM MgSO_4_, 20 mM HEPES, 5 mM NaOH, 1 mM (Na)PO_4_, 25 mM D-glucose

NH_4_Cl media: 25 mM NH_4_Cl, 105 mM NaCl, 2 mM CaCl_2_, 1 mM MgSO_4_, 20 mM HEPES, 8 mM NaOH, 5 mM KCl, 1 mM (Na)PO_4_, 25 mM D-glucose

TMA media: 130 mM TMA-Cl, 5 mM KCl, 2 mM CaCl_2_, 1 mM MgSO_4_, 20 mM HEPES, 8 mM TMA-OH, 1 mM (TMA)PO_4_, 25 mM D-glucose

### Electrophysiology

Electrophysiological experiments were conducted using a modified Ussing chamber^22,36^. Mouse jejunum and transplanted HIOs were dissected and immediately placed in ice-cold Krebs-Ringer solution. Tissues were opened to create a flat epithelial surface. Because seromuscular stripping is associated with release of cyclooxygenases and prostaglandins^22^, and prostaglandins can stimulate L-cells to release GLP1, GLP2 and PYY^37^, we performed the Ussing chamber experiments in intestinal tissue with an intact muscular layer. Tissues were mounted into sliders (0.031 cm^2^ area slider, P2307, Physiological Instruments) and placed in an Ussing chamber with reservoirs containing 5 mL buffer (115 mM NaCl, 1.2 mM CaCl_2_, 1.2 mM MgCl_2_, 25 mM NaHCO_3,_ 2.4 mM K_2_HPO_4_ and 0.4 mM KH_2_PO_4_). The mucosal and serosal tissue surfaces were bathed in the same solution, with the exception of 10 mM glucose in the serosal buffer and 10 mM mannitol in the luminal buffer. Mucosal and serosal reservoir solutions were gassed with 95% O_2_ and 5% CO_2_ to pH 7.4 and maintained at 37 °C by a circulating water bath behind the reservoir chambers. Tissue was allowed to equilibrate to a basal steady-state for a minimum of 30 min before the addition of chemicals or peptides. 10 nM tetrodotoxin (Tocris) was added to the serosal buffer bathing mouse intestine to inhibit voltage-gated neuronal firing, and allowed to incubate for a minimum of 10 min before basal *I*_sc_ recording. D-glucose and Gly-Sar were added to the luminal side of the chamber once the VIP-induced *I*_sc_ had stabilized at a maximum value.

**Table:** 

Tetrodotoxin	Tocris	10 nM
BIBO3304 trifluoroacetate	Tocris	300 nM
VIP	Tocris	10 nM
PYY(1–36)	Phoenix Pharmaceuticals	10 nM
CFTR(inh)-172	Millipore Sigma	20 μM
D-glucose	Sigma Aldrich	25 mM
Gly-Sar	Sigma Aldrich	20 mM

### Nutrient uptake assays

Transplanted HIOs were removed from the murine kidney, bisected to expose the lumen, and incubated with 100 mM 6-(*N*-(7-Nitrobenz-2-oxa-1,3-diazol-4-yl)Amino)−2-Deoxyglucose (6-NBDG) (Life Technologies) in 10 nM Tris/HEPES buffer containing 150 mM KCl or 150 mM NaCl for 30 min at 37 °C. Tissues were washed with ice-cold 10 mM Tris/HEPES buffer, then dissociated to single-cell suspension in 5 mL Tryple Select (Gibco) + 10 μM Y-27632 (Tocris), filtered, and subjected to analysis by flow cytometry.

HIOEs were differentiated for 5–7 days, then were removed from Matrigel and enzymatically dissociated into single-cell suspension using 0.25% Trypsin-EDTA. For Sodium Green, each cell preparation was split into two samples: one incubated with 25 mM D-glucose and one incubated in the absence of glucose. Each sample was incubated in Live Cell Imaging Solution (Invitrogen) containing 5 μM final concentration of Sodium Green tetraacetate (Molecular Probes) for 30 min at 37 °C, washed with ice-cold PBS and analyzed by flow cytometry. For dipeptide uptake assays, cell preparations were incubated with 100 µM β-Ala-Lys-AMCA (Biotrend) or 200 µM FITC-Gly-Sar (custom preparation from Pepscan, NL) for 30 min at 37 °C, washed with ice-cold PBS and analyzed by flow cytometry.

Undifferentiated enteroids that were “ready to split” were dissociated into single-cells and plated on transwell inserts (Corning)^38^, coated with Collagen IV (Sigma Aldrich). 300,000 cells were plated per 6.5 mm transwell insert. Differentiation was initiated at 24 h post-plating and monolayers were analyzed after 5–7 days. For glucose uptake, 1 mM fluorescent glucose analog 2-(*N*-(7-Nitrobenz-2-oxa-1,3-diazol-4-yl)Amino)−2-Deoxyglucose (2-NBDG, Life Technologies) was diluted in Live Cell Imaging Solution (Invitrogen) containing 25 mM D-glucose, and added to the apical surface of HIOE monolayers. For dipeptide uptake, 200 µM FITC-Gly-Sar (custom preparation by Pepscan, NL) was diluted in Live Cell Imaging Solution and added to the apical surface of the HIOE monolayer. The fluorescence intensity of Live Cell Imaging Solution in the basal chamber was quantified after 30 min at 37 °C. Intact barrier function was confirmed by co-incubation, quantification and exclusion of Cascade Blue conjugated 3000 MW dextran (Life Technologies) in every experiment. Monolayers were then excised from the plastic Transwell insert and mounted on a glass slide for live confocal Z-stack imaging using a Nikon A1 GaAsP LUNV inverted confocal microscope and NIS Elements software (Nikon).

### Intracellular pH assa**y**

Enteroids were differentiated for 5–7 days in the presence of vehicle (water or DMSO), 10 nM VIP (Tocris), 10 nM PYY (Phoenix Pharmaceuticals) and/or 300 nM BIBO3304. On the final day, enteroids were removed from Matrigel and enzymatically dissociated into single-cell suspension using 0.25% Trypsin-EDTA. Cell suspensions were counted and equal cell numbers of dissociated HIOEs were incubated in pHrodo Green AM Intracellular pH indicator (ThermoFisher Scientific) according to manufacturer’s directions for 30 min at 37 C, washed with 1X PBS, and analyzed by flow cytometry.

### Flow cytometry

After mechanical and enzymatic dissociation, tissues were filtered through a 40 μm cell strainer to obtain a single-cell suspension. In all experiments, samples were labeled with either CDH1-mRuby2 or Anti-EpCam-APC (BD Biosciences) to distinguish epithelial cells and incubated with SYTOX Blue dead cell stain (Life Technologies) or 7-AAD (BD Pharmingen). Forward scatter and side scatter were used to discriminate doublets and cellular debris. A minimum of 50,000 events per sample was recorded using an LSR Fortessa flow cytometer (BD Biosciences) and data were analyzed using FACSDiva software (BD Biosciences).

### Mice

B6.Cg-*Tg(Vil1-cre)*^*997Gum/J*^ (*VillinCre)* (JAX stock 004586)*, Neurog3*^*flox/flox*^^3^ and B6.Cg-*Gt(ROSA)26Sor*^*tm9(CAG−tdTomato)Hze*^/J (tdTomato)^39^ mice were maintained on a C57BL/6 background. Mice were housed in a specific pathogen-free barrier facility in accordance with NIH Guidelines for the Care and Use of Laboratory Animals. All experiments were approved by the Cincinnati Children’s Hospital Research Foundation Institutional Animal Care and Use Committee (IACUC2019-0006) and carried out using standard procedures. Mice were maintained on a 12 h light/dark cycle and had ad libitum access to standard chow and water. Mice were housed at 72 °F at 30–70% humidity. *VillinCre;Neurog3*^*flox/flox*^ mice^3^ and their littermates were weighed, genotyped and visually examined for liquid feces daily beginning at postnatal day 10. We established a diarrhea score, with 3 representing wet, yellow feces that smeared the perianal fur, and 0 representing normal, dry, brown, well-defined pellets. Mutant mice which suffered from diarrhea score 3 were included in the rescue experiment. 10 μg PYY (Phoenix Pharmaceuticals) was diluted in water and added to 20 μl DPP4 inhibitor (Millipore) to a final volume of 100 μl per mouse. Mice were injected intraperitoneally with this cocktail within 2 h of the onset of the dark cycle (7 pm) daily until analysis at postnatal day 28–35. Mice were given access to solid chow on the floor of the cage beginning at postnatal day 10 and weaned at postnatal day 21. Small intestinal transit was determined by oral gavage of food coloring diluted in 100 μl water to ad-lib fed mice, then sacrifice and measurement of the distance traveled by the dye-front 30 min post-gavage.

NSG mice hosting HIOs were treated with 25 μg PYY (Phoenix Pharmaceuticals) diluted in water to 100 μL by intraperitoneal injection. Mice were treated daily for a minimum of 10 days after HIOs had been maturing for 8 weeks, then dissected and analyzed.

### Statistics

Data are presented as the mean ± SEM unless otherwise indicated. Data represents measurements taken from individual mice and biological replicates of HIOs from two human pluripotent stem cell lines. Enteroid experiments were conducted on three independent lines. Significance was determined using appropriate tests in Graph Pad Prism, with *P* > 0.05 not significant; *P < 0.05, **P < 0.01, ***P < 0.001, ****P < 0.0001.

### Reporting summary

Further information on research design is available in the Nature Research Reporting Summary linked to this article.

## Supplementary information

Supplementary Information

Reporting Summary

## Data Availability

All data generated or analyzed during this study are included in the published article (and Supplementary Information files). Source data are available in the Source Data file, and available upon reasonable request from the corresponding author.

## Source data

Source Data
