# Supplementary material for: Enteroendocrine cells couple nutrient sensing to nutrient absorption by regulating ion transport
Source: Nat Commun. 2020 Sep 22;11:4791. doi: 10.1038/s41467-020-18536-z (PMC7508945; doi:10.1038/s41467-020-18536-z)
Supplement: Supplementary file 1 — Supplementary Information [file 41467_2020_18536_MOESM1_ESM.pdf]

Enteroendocrine cells couple nutrient sensing to nutrient absorption by regulating ion transport

McCauley, et al.

Supplementary Figures

McCauley et al Supplementary Figure 1

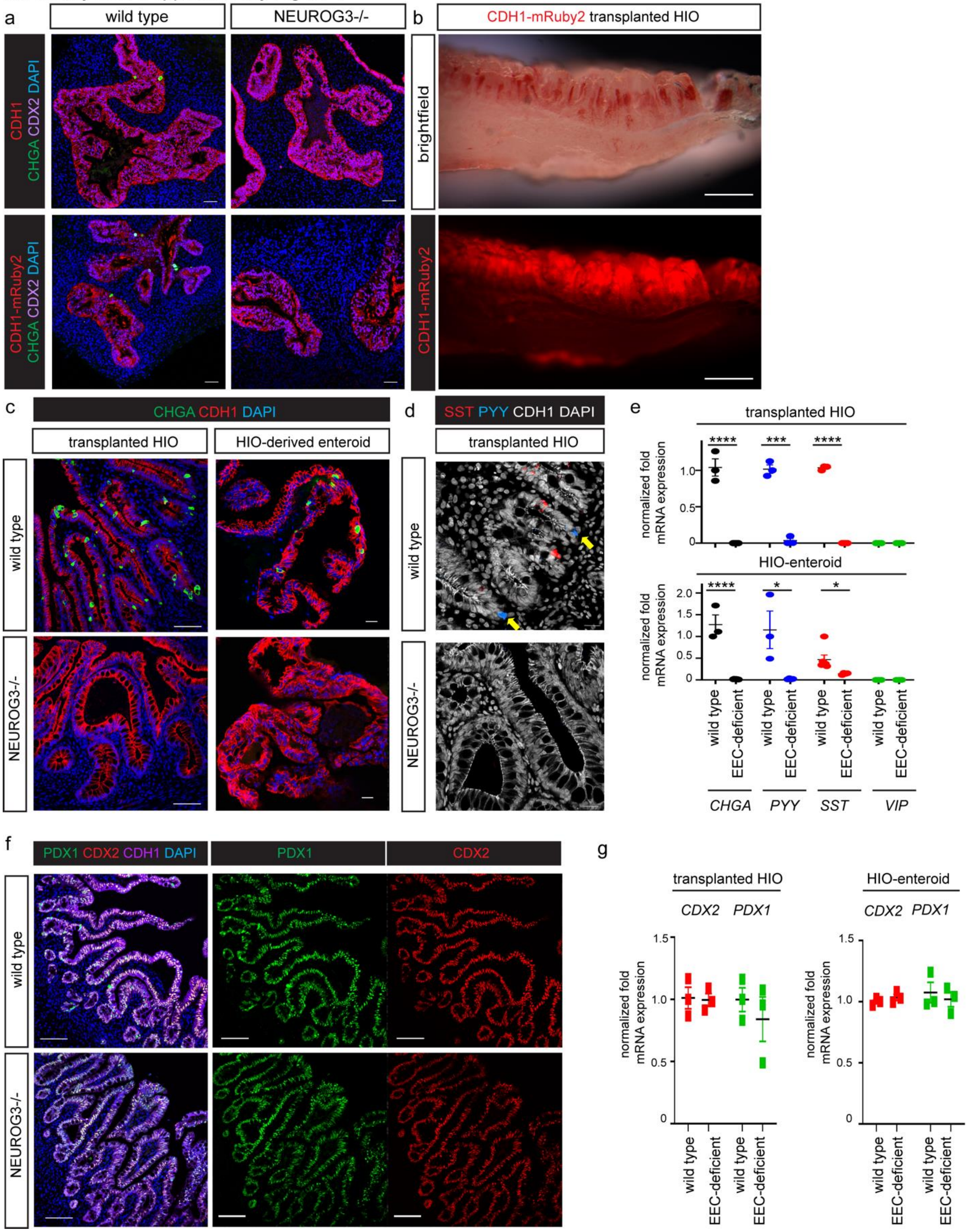

Supplementary Figure 1. NEUROG3 is required for enteroendocrine cell development in human intestinal organoids.

a. Human intestinal organoids (HIOs) derived from human pluripotent stem cells with a null mutation in *NEUROG3* lacked enteroendocrine cells (EECs) but otherwise had a normal morphology. The epithelial morphology was assessed using a PSC line expressing a CDH1-mRuby2 fusion protein[33] (red, bottom panels) and by co-staining with an anti-CDH1 antibody (red, top panels). Loss of NEUROG3 did not alter markers of intestinal identity (CDX2, purple). Only wild-type (top) and wild-type CDH1-mRuby2 (bottom) HIOs generated Chromogranin A (CHGA)- expressing EECs (green). Images shown are representative of over 20 experiments. Scale bars = 50 μm.

b. After maturation *in vivo*, HIOs develop well-defined crypt-villus architecture. Transplantation of HIOs (~1 mm) into mice for 10-12 weeks results in growth (1-2 cm), morphogenesis and maturation[16]. The epithelium is labeled by CDH1-mRuby2. Images shown are representative of 20 experiments. Scale bar = 500 μm.

c. Transplanted HIOs with disrupted *NEUROG3* lacked EECs as marked by CHGA+, but were otherwise morphologically normal. Scale bars = 100 μm. Enteroids derived from the crypts of transplanted HIOs produced EECs when differentiated, whereas those derived from EEC-deficient HIOs never did. Images shown are representative of 6 experiments. DAPI and CDH1 mark nuclei and epithelium, respectively. Scale bars = 20 μm.

d. Transplanted HIOs generated PYY+ (arrows) and somatostatin (SST)+ EECs, which were never detected in NEUROG3-deficient transplanted HIOs. DAPI and CDH1 mark nuclei and epithelium, respectively. Images are representative of 12 organoids. Scale bars = 50 μm.

e. EEC-deficient transplanted HIOs (top) and EEC-deficient HIO-derived enteroids (bottom) did not express mRNA for EEC markers *CHGA* (****P<0.001), *PYY* (***P=0.001) or *SST* (****P<0.0001). Neither wild-type nor EEC-deficient tissues expressed mRNA for *VIP* (n=9).

Data represents three biologically independent organoids and three independent enteroids lines per genotype. Statistics calculated by unpaired, two-tailed Student's *t*-test.

f. Regional patterning of transplanted HIOs was independent of NEUROG3. Transplanted HIOs, with and without EECs, coexpressed CDX2 and the proximal small intestinal marker PDX1. DAPI and CDH1 mark nuclei and epithelium, respectively. Images shown are representative of 20 experiments. Scale bars = 100 μm.

g. Regional identity of transplanted HIOs was maintained in enteroid culture. There was no difference in *CDX2* or *PDX1* mRNA expression between wild-type and EEC-deficient transplanted HIOs, or between wild-type and EEC-deficient HIO-derived enteroids. Data represents three enteroids lines generated from independent transplanted organoids. Statistics calculated by unpaired, two-tailed Student's *t*-test.

Error bars are ± SEM.

McCauley et al Supplementary Figure 2

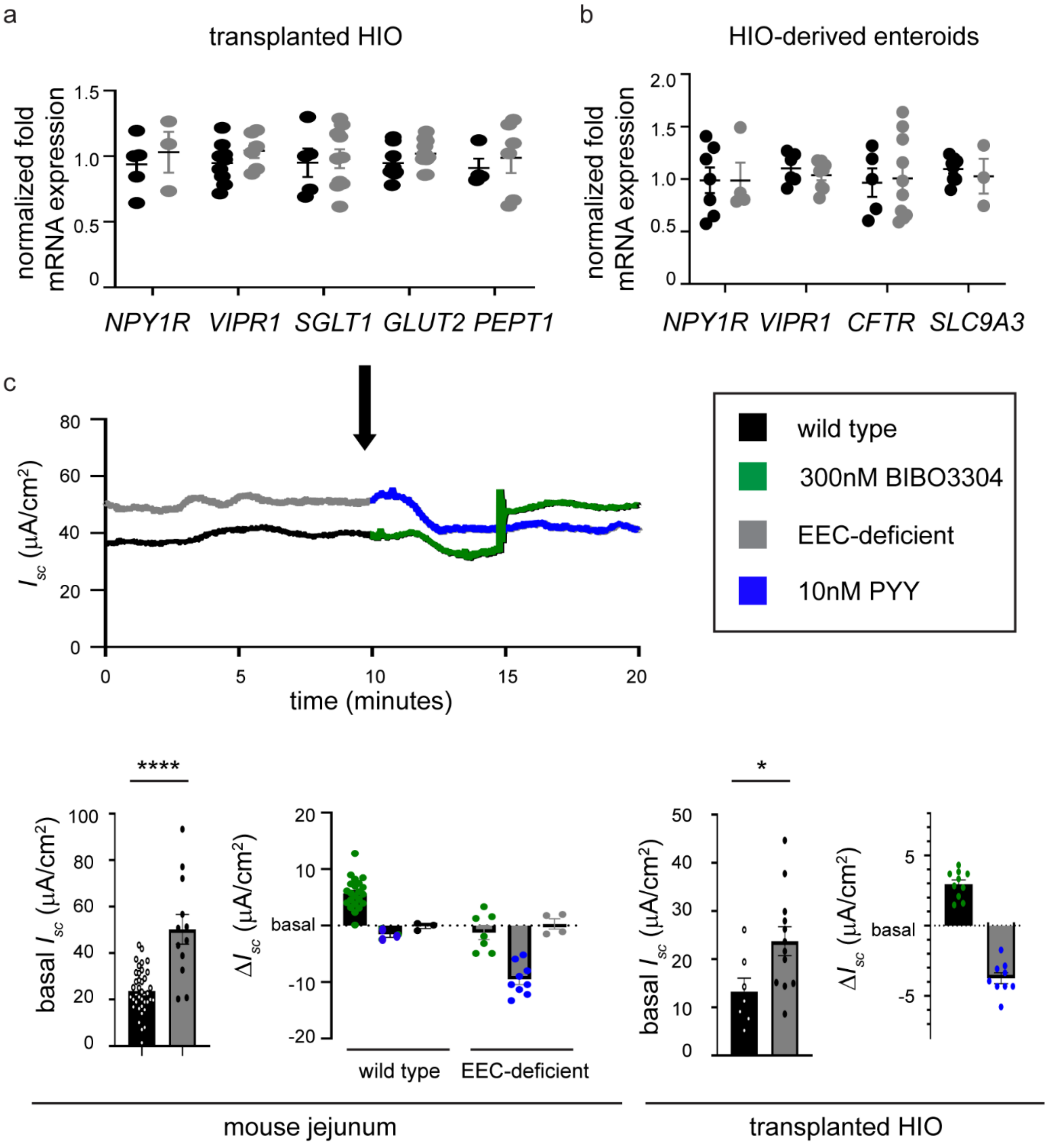

Supplementary Figure 2. PYY is required to maintain normal electrophysiology in mouse and human small intestine.

a. There was no difference in *NPY1R, VIPR1, SGLT1, GLUT2,* or *PEPT1* mRNA expression between transplanted HIOs with EECs and those without EECs. Data represents 4-6 biologically independent organoids per genotype.

b. There was no difference in *NPY1R, VIPR1, CFTR* or *SLC9A3* mRNA expression between enteroids generated from wild-type or EEC-deficient HIOs. Data represents cells obtained from 5-6 independent passages per genotype.

c. PYY modulates basal I_{sc} in human and mouse small intestine. EEC-deficient mouse and human small intestine had significantly higher basal I_{sc} than wild-type (mouse, n=36 wild-type, n=11 mutant, ****P<0.0001; HIO, n=7 wild-type, n=12 mutant, *p=0.03) after equilibration in the Ussing chamber. Addition of 300 nM NPY1R inhibitor BIBO3304 to wild-type tissues reproducibly increased the basal I_{sc} (mouse, n= 26, human, n=10), whereas addition of 10 nM PYY lowered the basal I_{sc} in mutant mouse and human tissue (mouse, n=9, human, n=9). Blocking NPY1R with BIBO3304 abolished the effect of PYY in both wild-type and mutant tissues. Arrow indicates time of PYY or BIBO3304 application to the experiment. One representative trace is shown (mouse). Statistics calculated by unpaired, two-tailed Student's *t*-test.

Error bars are ± SEM.

McCauley et al Supplementary Figure 3

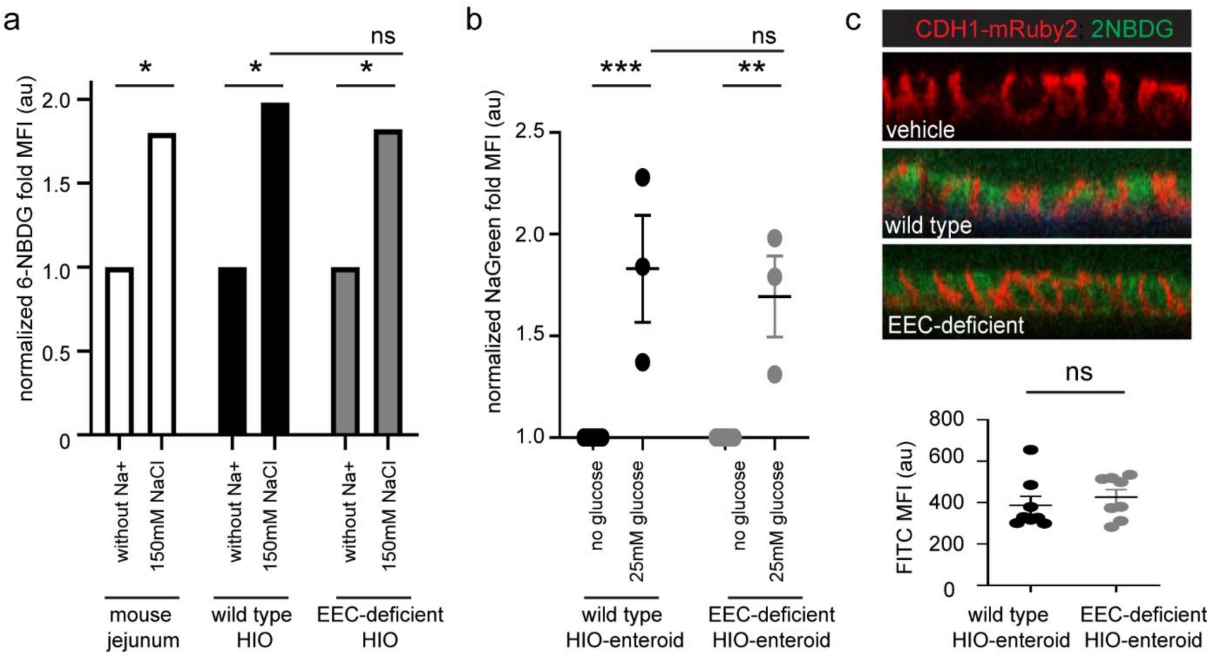

Supplementary Figure 3: SGLT1 is functional in EEC-deficient human small intestinal organoids.

a. EEC-deficient human small intestinal organoids displayed similar total uptake of the glucose analog 6-NBDG in the presence of NaCl (*P=0.01) as wild-type organoids (*P=0.01) and wild-type mouse jejunum cells (*P=0.01), demonstrating functional SGLT1-mediated transport. Data represents pooled samples from two independent biological replicates. Statistics calculated by two-way ANOVA with Tukey's multiple comparisons test.

b. The ability of SGLT1 to transport Na^+ in the presence of glucose was not altered in EEC-deficient enteroids (***P=.0005, wild type, **P=.0023, EEC-deficient). Data represents mean fluorescence intensity (MFI) of the fluorescent indicator NaGreen in 3 independent experiments. Statistics calculated by two-way ANOVA with Tukey's multiple comparisons test.

c. Total glucose transport is similar in wild-type and EEC-deficient enteroid monolayer cultures. The fluorescence intensity of the basal chamber was quantified after 30 minutes of exposure to 25 mM D-glucose with 1 mM fluorescent glucose analog 2-NBDG on the apical surface (lower graph). The epithelium was then visualized for 2-NBDG within CDH1-mRuby2-positive epithelium. Data represents 8 independent experiments. Statistics calculated by unpaired, two-tailed t-test.

All error bars are ± SEM.

McCauley et al Supplementary Figure 4

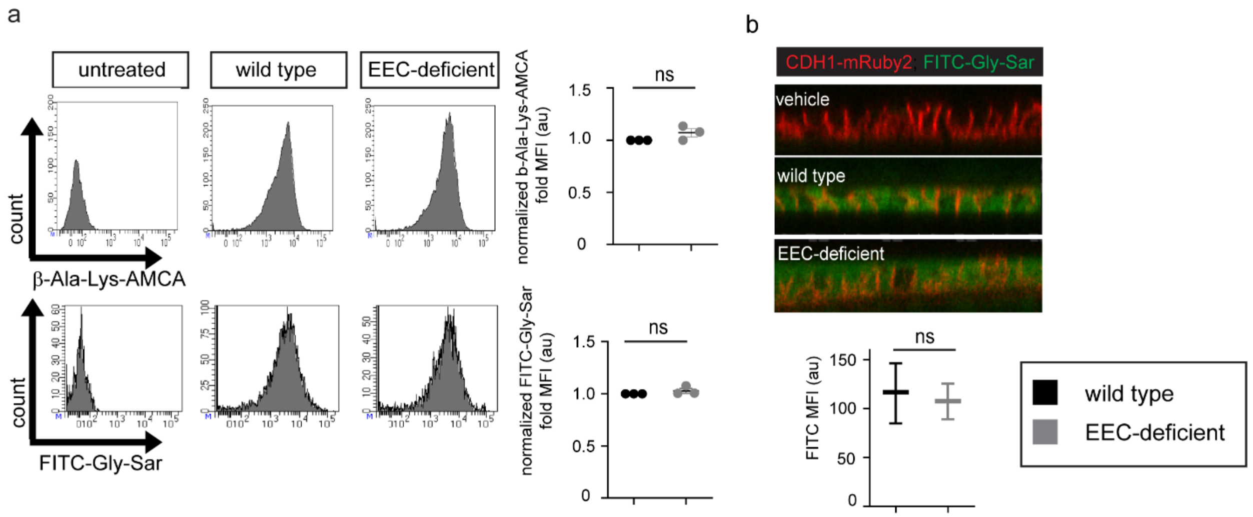

Supplementary Figure 4. EEC-deficient epithelium is competent to absorb dipeptides *in vitro.*

a. Wild-type and EEC-deficient enteroids were differentiated for 5-7 days then exposed to 100 µM β-Ala-Lys-AMCA (top) or 200 µM FITC-Gly-Sar (bottom). Enteroids were dissociated and analyzed by flow cytometry. Histograms represent live, EpCam+ epithelial cells after debris exclusion and doublet discrimination. Scatter plots represent mean fluorescence intensity (MFI) normalized to wild-type. n=3 independent experiments. Statistics calculated by unpaired, two-tailed t-test.

b. Total dipeptide transport was similar between wild-type and EEC-deficient enteroid monolayer cultures. The fluorescence intensity of the basal chamber was quantified after 30 minutes of exposure to 200 µM FITC-Gly-Sar on the apical surface (lower graph). The epithelium was then visualized for FITC fluorescence within CDH1-mRuby2-positive epithelium. Data represents 3 independent experiments. Statistics calculated by unpaired, two-tailed t-test.

All error bars are ± SEM.

McCauley et al Supplementary Figure 5

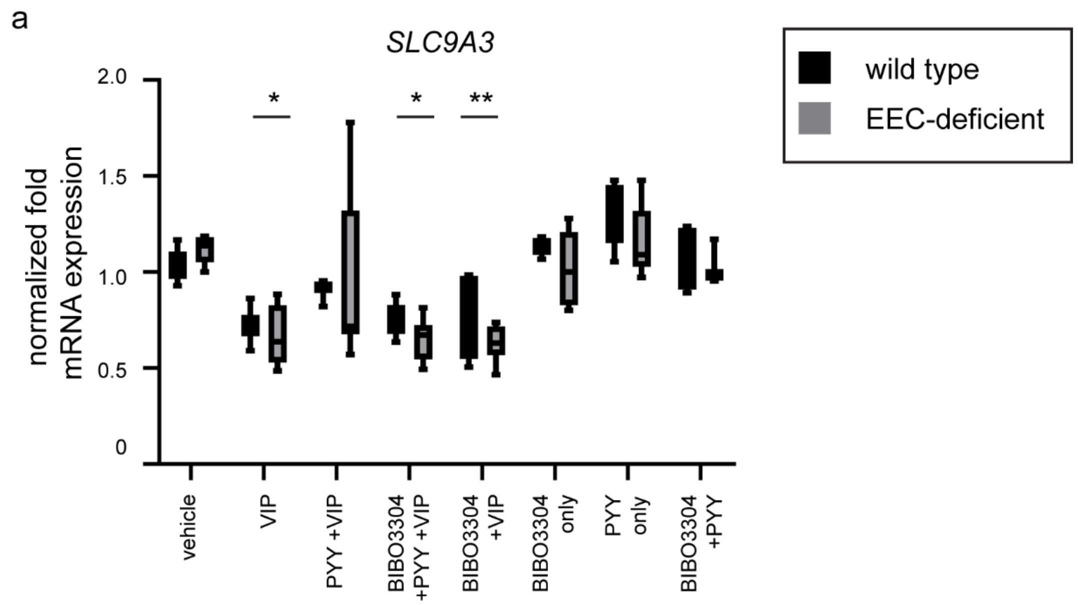

Supplementary Figure 5. VIP and PYY regulate NHE3 expression in human small intestinal epithelium.

a. The PYY-VIP axis regulates *SLC9A3* expression. After 5-7 days of exposure to VIP, *SLC9A3* expression was reduced in wild-type and in EEC-deficient enteroids (*P=0.04). Exposure to PYY concurrently with VIP restored *SLC9A3* expression to not significantly different from untreated in both groups. The effect of PYY was blocked with the NPY1R inhibitor BIBO3304 (*P=0.02). While there was a trend for PYY treatment alone to increase *SLC9A3* expression, this did not reach significance. n=6 independent experiments. Error bars are + SEM; statistics calculated by two-way ANOVA with Tukey's multiple comparisons test.

McCauley et al Supplementary Figure 6

a

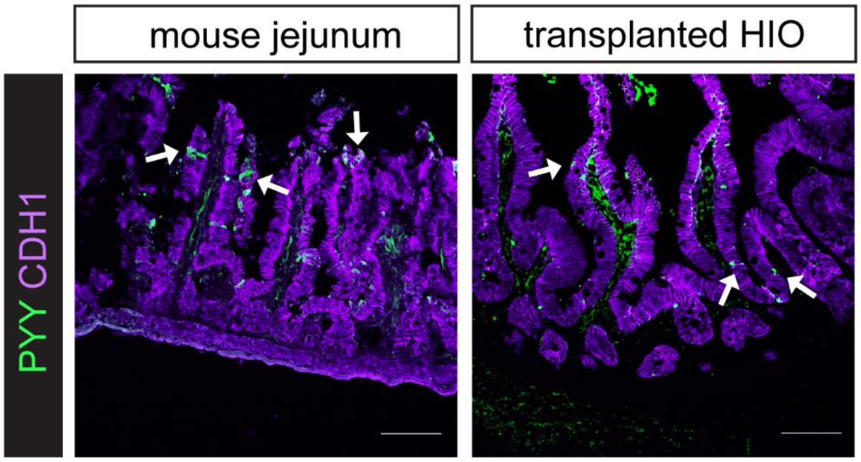

Supplementary Figure 6. PYY is abundant in mouse and human small intestine.

a. PYY+ EECs (arrows) are abundant in mouse and human small intestine. CDH1 labels epithelium in purple. Images are representative of 8 independent mice and transplanted human intestinal organoids. Scale bars = 100 μM.

McCauley et al Supplementary Figure 7

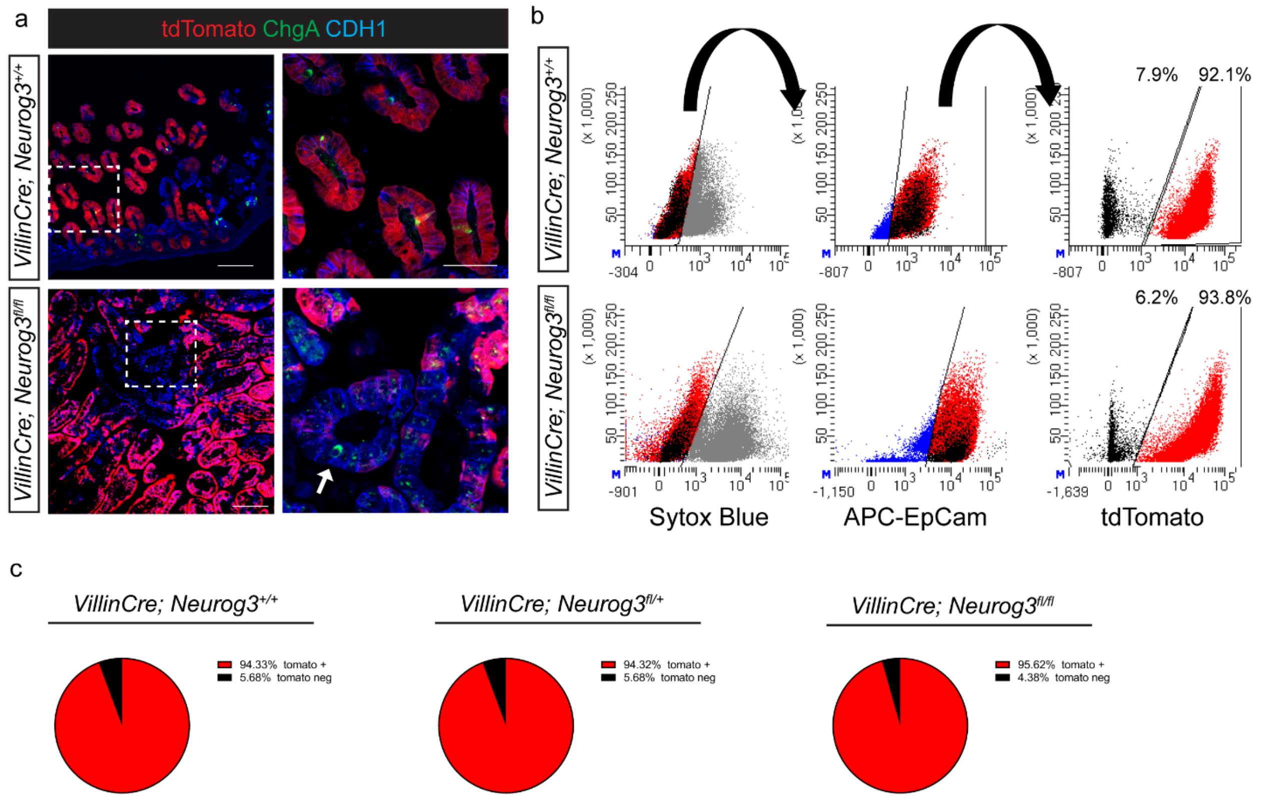

Supplemental Figure 7. *VillinCre; Neurog3$^{flox/flox}$; Rosa26$^{Flox\text{-}STOP\text{-}flox\text{-}tdTomato}$* mice display incomplete recombination.

a. The tdTomato reporter revealed regions of jejunal epithelium that escaped recombination by *VillinCre.* ChgA+ EECs were abundant in tdTomato+ and tdTomato negative regions of wild-type jejunum, but were only detected in tdTomato negative epithelium of *Neurog3$^{fl/fl}$* animals (arrow). Images are representative of 3 wild-type and 3 mutant mice. Scale bars = 20μm.

b. Representative dot plots and gating strategy from flow cytometric analysis of *VillinCre; Neurog3$^{+/+}$; Rosa26$^{Flox\text{-}STOP\text{-}flox\text{-}tdTomato}$* and *VillinCre; Neurog3$^{flox/flox}$; Rosa26$^{Flox\text{-}STOP\text{-}flox\text{-}tdTomato}$* jejunum.

c. Quantification of efficiency of recombination of *VillinCre*. Jejunum of *VillinCre; Neurog3$^{+/+}$; Rosa26$^{Flox\text{-}STOP\text{-}flox\text{-}tdTomato}$*, *VillinCre; Neurog3$^{fl/+}$; Rosa26$^{Flox\text{-}STOP\text{-}flox\text{-}tdTomato}$* and *VillinCre; Neurog3$^{fl/fl}$; Rosa26$^{Flox\text{-}STOP\text{-}flox\text{-}tdTomato}$* were subjected to flow cytometry. After doublet discrimination, live, EpCam^{+} cells were analyzed for tdTomato expression. Approximately 5.675 $\pm$ 1.98% of wild-type (n=8), 5.678 $\pm$ 3.2% of heterozygous (n=9), and 4.38 $\pm$ 2.56% of mutant jejunum (n=5) escaped labeling with the tdTomato reporter.

McCauley et al Supplementary Figure 8

a

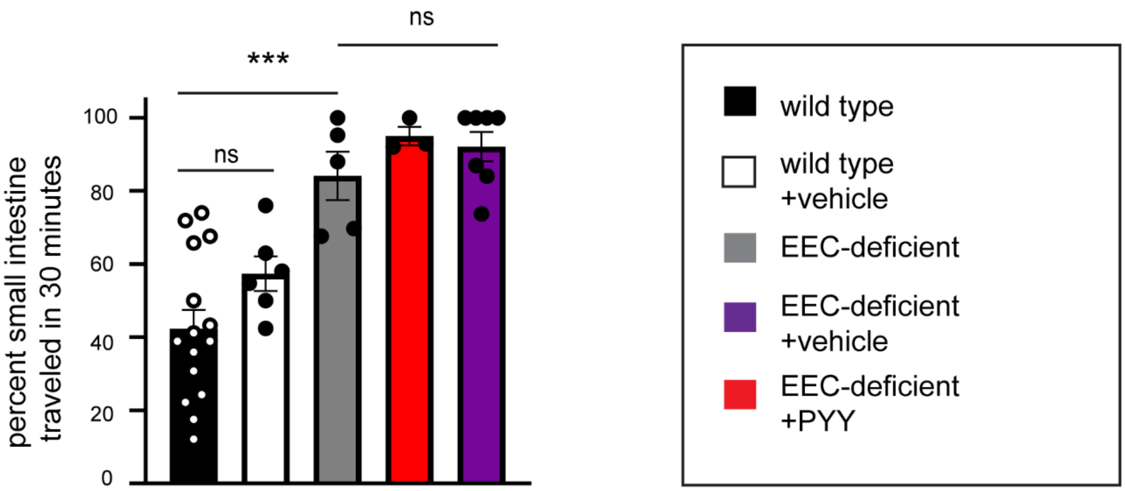

Supplemental Figure 8. PYY(1-36) does not slow intestinal motility in EEC-deficient mice.

a. The mechanism of improved survival and diarrhea in PYY-treated mutant animals does not include slowing intestinal motility. Animals fed ad-lib were orally gavaged with dye-colored water then sacrificed 30 minutes later. The distance traveled by the dye-front was reported as percent of small intestinal length. n=15 wild-type mice, 6 wild-type + vehicle mice, 5 mutant mice (***P=0.0002), 3 mutant + PYY mice, 7 mutant + vehicle mice. Error bars are $\pm$ SEM. Statistics calculated by one-way ANOVA with Tukey’s multiple comparisons test.
