# Supplementary material for: Enteroendocrine cells couple nutrient sensing to nutrient absorption by regulating ion transport
Source: Nat Commun. 2020 Sep 22;11:4791. doi: 10.1038/s41467-020-18536-z (PMC7508945; doi:10.1038/s41467-020-18536-z)
Supplement: Supplementary file 2 — Reporting Summary [file 41467_2020_18536_MOESM2_ESM.pdf]

Corresponding author(s): James M. Wells

Last updated by author(s): Jul 24, 2020

Reporting Summary

Nature Research wishes to improve the reproducibility of the work that we publish. This form provides structure for consistency and transparency in reporting. For further information on Nature Research policies, see our Editorial Policies and the Editorial Policy Checklist.

Statistics

For all statistical analyses, confirm that the following items are present in the figure legend, table legend, main text, or Methods section.

n/a	Confirmed	
☐	☒	The exact sample size (*n*) for each experimental group/condition, given as a discrete number and unit of measurement
☐	☒	A statement on whether measurements were taken from distinct samples or whether the same sample was measured repeatedly
☐	☒	The statistical test(s) used AND whether they are one- or two-sided<br>*Only common tests should be described solely by name; describe more complex techniques in the Methods section.*
☒	☐	A description of all covariates tested
☒	☐	A description of any assumptions or corrections, such as tests of normality and adjustment for multiple comparisons
☐	☒	A full description of the statistical parameters including central tendency (e.g. means) or other basic estimates (e.g. regression coefficient) AND variation (e.g. standard deviation) or associated estimates of uncertainty (e.g. confidence intervals)
☐	☒	For null hypothesis testing, the test statistic (e.g. *F*, *t*, *r*) with confidence intervals, effect sizes, degrees of freedom and *P* value noted<br>*Give P values as exact values whenever suitable.*
☒	☐	For Bayesian analysis, information on the choice of priors and Markov chain Monte Carlo settings
☒	☐	For hierarchical and complex designs, identification of the appropriate level for tests and full reporting of outcomes
☒	☐	Estimates of effect sizes (e.g. Cohen's *d*, Pearson's *r*), indicating how they were calculated

Our web collection on statistics for biologists contains articles on many of the points above.

Software and code

Policy information about availability of computer code

Data collection	NIS Elements AR 5.20.00 (Nikon)<br>FACSDiva Version 8.0.2 (BD Biosciences)<br>QuantStudio Design & Analysis v1.4.3 (Applied Biosystems)<br>Acquire and Analyze 2.3.8 (Physiologic Instruments)
Data analysis	Microsoft Excel 2016<br>GraphPad Prism 8

For manuscripts utilizing custom algorithms or software that are central to the research but not yet described in published literature, software must be made available to editors and reviewers. We strongly encourage code deposition in a community repository (e.g. GitHub). See the Nature Research guidelines for submitting code & software for further information.

Data

Policy information about availability of data

All manuscripts must include a data availability statement. This statement should provide the following information, where applicable:

- Accession codes, unique identifiers, or web links for publicly available datasets
- A list of figures that have associated raw data
- A description of any restrictions on data availability

All data generated or analyzed during this study are included in the published article (and supplementary information files).

Field-specific reporting

Please select the one below that is the best fit for your research. If you are not sure, read the appropriate sections before making your selection.

- [x] Life sciences
- [ ] Behavioural & social sciences
- [ ] Ecological, evolutionary & environmental sciences

For a reference copy of the document with all sections, see nature.com/documents/nr-reporting-summary-flat.pdf

Life sciences study design

All studies must disclose on these points even when the disclosure is negative.

Sample size	Sample sizes were not predetermined. Sample sizes differed based on the type of experiment and source material (ie mouse, organoid or enteroid). For mouse experiments, sample size ranged from 2-100 individual mice. For transplanted organoid experiments, sample size ranged from 3-20 individual grafts. For enteroid experiments, sample size ranged from 6 to 351 individual structures.
Data exclusions	Data were only excluded due to technical malfunction or human error.
Replication	All experiments were replicated using independently generated human intestinal organoid tissues, at least two enteroid cell lines, and at least two mutant mice from independent litters with littermate controls. All experiments were performed at least three times unless otherwise stated. All attempts at replication were successful.
Randomization	Samples were randomly organized into experimental groups based on availability of suitable control samples and timing of experiments. There was no predetermined allocation of samples to experimental groups.
Blinding	Investigators were blinded when quantifying wild-type versus EEC-deficient samples. When obtaining data, investigators were not blinded.

Reporting for specific materials, systems and methods

We require information from authors about some types of materials, experimental systems and methods used in many studies. Here, indicate whether each material, system or method listed is relevant to your study. If you are not sure if a list item applies to your research, read the appropriate section before selecting a response.

Materials & experimental systems

n/a	Involved in the study	
[ ]	[x]	Antibodies
[ ]	[x]	Eukaryotic cell lines
[x]	[ ]	Palaeontology and archaeology
[ ]	[x]	Animals and other organisms
[x]	[ ]	Human research participants
[x]	[ ]	Clinical data
[x]	[ ]	Dual use research of concern

Methods

n/a	Involved in the study	
[x]	[ ]	ChIP-seq
[ ]	[x]	Flow cytometry
[x]	[ ]	MRI-based neuroimaging

Antibodies

Antibodies used

CDX2: BioGenex #cdx2-88
CDX2: Cell Marque (Sigma) #EPR2764Y
CHGA: DHSB #CPTC-CHGA-1
CHGA: Immunostar #20086
CDH1: R&D #AF648
GLUT2: Santa Cruz #sc-7580
MUC2: Santa Cruz #sc-15334
NPY1R: Abcam #ab91262
PDX1: Abcam #ab47383-100
PEPT1: Santa Cruz #sc-20653
PYY: Abcam #ab22663
SGLT1: Santa Cruz #sc-98974
Somatostatin: Santa Cruz #sc-7819
VIPR1: ThermoFisher Scientific #PA3-113
IgG (H+L) Highly Cross-Adsorbed Donkey anti-Mouse, Alexa Fluor® 647, Invitrogen #A31571
Donkey anti-Goat IgG (H+L) Cross-Adsorbed Secondary Antibody, Alexa Fluor 568, Invitrogen #A-11057
Alexa Fluor® 488 Donkey Anti-Rabbit IgG, Invitrogen #A21206
Alexa Fluor® 488 Donkey Anti-Goat IgG, Invitrogen #A11055

April 2020

	Alexa Fluor 546 Donkey Anti-Mouse, Invitrogen #A10036<br>Donkey anti-Rabbit IgG (H+L) Highly Cross-Adsorbed Secondary Antibody, Alexa Fluor 647, Invitrogen #A31573
Validation	CDX2, CDH1, MUC2, PDX1, PYY validated by IF in human intestinal tissue in Munera et al doi.org/10.1016/j.stem.2017.05.020.<br>GLUT2, Somatostatin validated by immunofluorescence on mouse pancreas in Sinagoga et al doi:10.1242/dev.146316<br>CHGA (DHSB) validated by IF in mouse intestine in Sun et al http://doi.org/10.1098/rsob.170256<br>CHGA (Immunostar) has been cited 53 times and is validated by IHC in rat adrenal medulla and rat stomach as listed on their website: https://www.immunostar.com/shop/antibody-catalog/sp-1-chromogranin-a-porcine-antibody<br>NPY1R validated by WB in human brain tissue lysate, human heart tissue lysate, human glioblastoma, Caco-2 cells, HeLa cells; and by immunofluorescence staining in SKNSH cells as listed on their website https://www.abcam.com/npy1r-antibody-ab91262.html<br>PEPT1 cited by 8 articles on their website and validated by IF in HEK293 cells in Xu et al http://doi.org/10.1007/s00232-010-9317-7<br>SGLT1 validated by immunofluorescence in A431 cells and IHC in lung fibrose tissue as listed on their website https://www.thermofisher.com/antibody/product/SGLT1-Antibody-Polyclonal/PA5-28240<br>VIPR1 validated by IHC on human intestine as listed on the website: https://www.thermofisher.com/antibody/product/VIPR1-Antibody-Polyclonal/PA3-113

Eukaryotic cell lines

Policy information about cell lines

Cell line source(s)	WiCell WA01
Authentication	The WA01 human embryonic stem cells (H1; NIH registration number 0043) used in this study were obtained from a cryopreserved distribution bank generated by the CCHMC Pluripotent Stem Cell Facility (PSCF). Cells were authenticated at the time of bank cryopreservation by analysis of karyotype, identity, mycoplasma contamination, and tri-lineage differentiation. Metaphase spread and G-banded karyotype analysis was performed in the CCHMC Cytogenetics Laboratory and cells were demonstrated to be karyotypically normal. Cell identity analysis was by STR analysis performed by Genetica DNA Laboratories (a LabCorp brand). Cells were demonstrated to match the Cellosaurus WA01 profile (https://web.expasy.org/cellosaurus/CVCL_9771) at all tested loci (CSF1PO, D3S1358, D5S818, D7S820, D8S1179, D13S317, D16S539, D18S51, D21S11, FGA, Penta D, Penta E, TH01, TPOX, vWA), confirming cell identity. Cells were confirmed to be mycoplasma-free by MycoAlert assay (Lonza). Trilineage differentiation potential was demonstrated with the Scorecard analysis (ThermoFisher). Following thaw from the distribution bank, the recovered cells were cultured for a maximum of 20 passages. After 20 passages, each culture was discarded and a replacement culture obtained from the bank.
Mycoplasma contamination	All cell lines tested negative for mycoplasma contamination.
Commonly misidentified lines<br>(See ICLAC register)	No commonly misidentified cell lines were used in this study.

Animals and other organisms

Policy information about studies involving animals; ARRIVE guidelines recommended for reporting animal research

Laboratory animals	This study involved Mus musculus on a C57Bl/6 background. Mice of both sexes were used equally from birth to approximately 6 weeks of age. B6.Cg-Tg(Vil1-cre)997Gum/J (VillinCre) (JAX stock 004586), Neurog3flox/flox and B6.Cg-Gt(ROSA)26Sortm9(CAG-tdTomato)Hze/J (tdTomato) mice were maintained on a C57BL/6 background and genotyped as previously described. Mice were housed in a specific pathogen free barrier facility in accordance with NIH Guidelines for the Care and Use of Laboratory Animals. Mice were maintained on a 12-hour light/dark cycle at 72 degrees F, 30-70% humidity, and had ad libitum access to standard chow and water. Mice were weaned at postnatal day 21 and housed at no more than 4 adults per cage.
Wild animals	This study did not use wild animals.
Field-collected samples	This study did not involve samples collected from the field.
Ethics oversight	All experiments were approved by the Cincinnati Children's Hospital Research Foundation Institutional Animal Care and Use Committee (IACUC2019-0006).

Note that full information on the approval of the study protocol must also be provided in the manuscript.

Flow Cytometry

Plots

Confirm that:

- [x] The axis labels state the marker and fluorochrome used (e.g. CD4-FITC).
- [x] The axis scales are clearly visible. Include numbers along axes only for bottom left plot of group (a 'group' is an analysis of identical markers).
- [x] All plots are contour plots with outliers or pseudocolor plots.
- [x] A numerical value for number of cells or percentage (with statistics) is provided.

Methodology

Sample preparation	Enteroids were removed from Matrigel, mechanically dissociated using ice-cold 0.3mM EDTA and enzymatically dissociated using 0.25% Trypsin-EDTA. Single-cell suspensions were filtered through a 40uM filter and equal numbers of cells aliquotted per tube according to the experimental design.<br>Full-thickness human intestinal organoid and mouse intestine were rinsed in ice-cold PBS, bisected to remove mucus and debris, and dissociated in Tryple (Gibco) containing Y-27632 dihydrochloride at 4C while shaking. After 45-60 minutes, the cell suspension was enzymatically dissociated with Accutase (STEMCELL Technologies) then filtered through a 40uM filter before aliquotting equal cell numbers to each tube according to the experimental design.
Instrument	Data were collected on the LSR Fortessa flow cytometer (BD Biosciences).
Software	Data were collected and analysed using FACSDiva software (BD Biosciences).
Cell population abundance	Negative controls and single-stained controls were used to confirm the abundance of the desired populations. Cells were not sorted in this study, so no post-sort analysis was conducted.
Gating strategy	Forward scatter and side scatter were used to discriminate doublets and cellular debris. A live/dead marker was always included and further populations gated only on live, single-cells. Gates were confirmed based on negative and single-stained controls.

☒ Tick this box to confirm that a figure exemplifying the gating strategy is provided in the Supplementary Information.
